# Performance of textured dual mobility total hip prosthesis with a concave dimple during Muslim prayer movements

**DOI:** 10.1038/s41598-023-50887-7

**Published:** 2024-01-09

**Authors:** M. Muchammad, Mohammad Tauviqirrahman, Muhammad Imam Ammarullah, Muhammad Iqbal, Budi Setiyana, J. Jamari

**Affiliations:** 1 Laboratory for Engineering Design and Tribology, Department of Mechanical Engineering, Universitas Diponegoro, Semarang, 50275 Central Java Indonesia; 2 Department of Mechanics and Aerospace Engineering, College of Engineering, Southern University of Science and Technology, Shenzhen, 518055 Guangdong China; 3 Biomechanics and Biomedics Engineering Research Centre, Universitas Pasundan, Bandung, 40153 West Java Indonesia; 4 Laboratory for Surface Technology and Tribology, Faculty of Engineering Technology, University of Twente, Postbus 217, 7500 AE Enschede, The Netherlands

**Keywords:** Engineering, Mechanical engineering

## Abstract

The single mobility bearing as a previous bearing design of total hip prosthesis has severe mobility constraints that can result in dislocation during Muslim (people who follow the Islam as religion) prayer movements, specifically shalat that requires intense movement. There are five intense movements (i.e., bowing, prostration, sitting, transition from standing to prostration, and final sitting) during Muslim prayer that may generate an impingement problem for patients with total hip prosthesis. In this work, textured dual mobility total hip prosthesis with two textured cases (i.e., textured femoral head and textured inner liner) are presented and their performances are numerically evaluated against untextured surface model during Muslim prayer movement. The concave dimple design is chosen for surface texturing, while for simulating femoral head materials, SS 316L and CoCrMo is choosen. To represent the real condition, three-dimensional computational fluid dynamics (CFD) coupled with two-way fluid–structure interaction (FSI) methods are employed to analyze elastohydrodynamic lubrication problem with non-Newtonian synovial fluid model. The main aim of the present study is to investigate the tribological performance on dual mobility total hip prosthesis with applied textured surface with concave dimple in femoral head and inner liner surface under Muslim prayer movements. It is found that applying surface texturing has a beneficial effect on the lubrication performance for some intense movements. The textured femoral head model performs better than textured inner liner model and untextured model (both femoral head and inner liner). The numerical results also indicate superior performance of CoCrMo femoral head compared to SS 316L femoral head. These findings can be used as a reference for biomedical engineers and orthopedic surgeons in designing and choosing suitable total hip prosthesis for Muslims makes they can carry out Muslim prayer movements like humans in general who have normal hip joints.

## Introduction

Total hip replacement is one of several effective orthopedic surgical techniques for the treatment of hip joint issues caused by trauma or osteoarthritis. The hip joint is shaped like a ball and socket, connecting the femur to the acetabulum^1^. During a total hip replacement, the damaged bone and cartilage are removed and replaced with prosthetic components^2^. It is estimated that total hip replacement will increased due to aging population with the old age group demanding for a higher quality of life^3^.

Numerous biomaterials are being developed for used in bearing of total hip prosthesis. Metals^4^, ceramics^5^, and polyethylene^6^ materials are frequently employed in implant preparation. Due to their excellent wear resistance, bearing combination such as metal-on-metal^7^, metal-on-polyethylene^8^, ceramic-on-ceramic^9^, and ceramic-on-polyethylene^10^ has been developed. Wear on bearing of total hip prosthesis, which is influenced by a number of contributing elements such as contact pressure^11^, lubrication^12^, and motions^13^ is believed to be one of the major reasons for implant failure. Numerous studies have been conducted for reducing wear on bearing of total hip prosthesis in order to achieve longer time used, where one of them effort is surface modification^14–16^. Laser surface engineering^17^ and electrical discharge maching^18^ are two popular approaches for surface modification of total hip prosthesis that texturing bearing interface to create dimple. Application of surface texturing on bearing of hip prosthesis with dimple on interacting surfaces would bring several advantages, such as reducing wear rate^19^, increasing lubrication performance^20^, and trapping wear debris^21^. Then, maximize dimple parameter such as depth^22^, diameter^23^, number^24^, pattern^25^, space^26^, and shape^27^ is crucial as explained be Pakhaliuk et al.^28^ for their study in textured femoral head of hip prosthesis.

Hip impingement as one of the main sources of hip joint failure can be caused by intense human movement like yoga as a type of Japanese style activity^29^ and Salat as a type of Muslim (people who follow the Islam as religion) praying activity^30^. Furthermore, based on a retrospective study by Anwar et al.^31^, 50% of patients in a total of 22 subjects with total hip prosthesis were still unable to complete Muslim prayer movements in an excellent manner postoperatively. As a result, some modification of movement is required duting Muslim prayer movements. On a orthopedic surgeon’s recommendation, people with total hip prosthesis are restricted from engaging in intense movement during such activities to prevent hip dislocation. Focusing on the development of the design of total hip prothesis that suitable for accommodate Muslim prayer movements, Saputra et al.^32–36^ have conducted several studies based on the numerical approach to reduce the potential impingement and risk of dislocation of the hip joint movement under intense conditions that founding the conclusion that bearing of total hip prosthesis with single mobility design cannot accommodate Muslim prayer movements. Then, dual mobility bearing as a new design for total hip prosthesis has been explored during Muslim prayer movements as presented in previous study by Wibowo et al.^37^ and Jamari et al. ^38^.

The development of dual mobility bearing of total hip prosthesis purposed to accommodate Muslim prayer movements can be carried out using clinical^39^, experimental^40^ and computational^41^ approaches. The clinical approach is the most realistic approach by creating a dual mobility total hip prosthesis that is used directly by Muslims to accommodate Muslim prayer movements. Unfortunately, a clinical approach will require complete patient commitment and participation, without which the results obtained will be invalid^42^. An experimental approach may be an option besides clinical testing using a hip joint simulator. However, the weakness of this method is that it requires a long time and adequate equipment^43^. A third approach using computational simulation can be a solution to the obstacles found from clinical and experimental approaches in efforts to develop dual mobility total hip prosthesis to accommodate Muslim prayer movements^44^.

This work aims to investigate the influence of surface texturing applied either on femoral head or liner for improving the tribological performance on the dual mobility total hip prosthesis during Muslim prayer movements. The focus will be on enhancing the load support indices under intense movements as found in Muslim praying activity based on computational fluid dynamics (CFD)–fluid–structure interaction (FSI) methods. To explore the benefits of texturing in the hip prosthesis, in the following computations, all textured cases are compared to the untextured surface. Furthermore, a fluid structural investigation of stainless steel 316L (SS 316L) and cobalt chrome molybdenum (CoCrMo) alloy femoral heads was also performed using two dimpled models. The load-carrying capacity, maximum principal stress, and deformation parameters for the design strategy of dual mobility total hip prosthesis during Muslim prayer movements are derived from the findings of computational simulation.

## Materials and method

### Governing equation

In this research, the synovial fluid behavior caused by hip motion is solved by simultaneously solving the continuity equation and the Navier–Stokes equation^45^. Furthermore, the elasticity equation is used to solve the calculation in the solid computational. For the simulation, the elastohydrodynamic condition was evaluated in both the fluid and solid domains using ANSYS 18.0. The expressions of the mass conservation equation and momentum conservation equation are respectively, given as follows:

1 $$\nabla \cdot V = 0$$

2 $$\rho \left( {V \cdot \nabla } \right)V = - \nabla p + \nabla \cdot \left( {\mu \nabla V} \right)$$

The FSI procedure establishes the coupling of fluid dynamics and structural mechanics. The solid domain is governed by structural dynamics equations and obeys Newton's second law as follows:

3 $$\rho_{s} {\mathbf{\ddot{d}}} = \nabla \cdot {{\varvec{\upsigma}}}_{{\mathbf{s}}} + {\mathbf{F}}_{{\mathbf{s}}}$$

The fluid and solid interact, causing the fluid pressure and the solid structure to be correlated in the bearing. The structure deforms due to the pressurized fluid. The deformed surface, in turn, causes a change in the lubricant domain. The displacement compatibility (kinematic condition) and stress equilibrium (dynamic condition) equations are created for data exchange between the fluid and the structure.

4 $${\mathbf{d}}_{f} = {\mathbf{d}}_{s}$$

5 $${\mathbf{n}} \cdot {{\varvec{\uptau}}}_{f} = {\mathbf{n}} \cdot {{\varvec{\uptau}}}_{s}$$

### Lubricant film thickness

The relative displacement that occurs on the surface of the fluid domain is proportional to the displacement that occurs in the solid domain. On the basis of the elastic deformation that occurs, the thickness of the film can be expressed in the form:

6 $$h\,\, = \,\,c\, + \,\Delta h\, + \,\delta$$

where *h* is the relative rigid displacement of the two contacting surfaces, *c* is the clearance between the femoral head and the acetabular liner, and *δ* is the total elastic deformation of the two contacting surfaces.

### Viscosity modeling in synovial fluid

In the synovial joint cavity, synovial fluid is a viscous than modelled ad non-Newtonian fluid^46^. It is classified as a non-Newtonian fluid because its viscosity may fluctuate from liquid to dense depending on the shear rate. Another study by Yao et al.^47^ stated that synovial fluid has a large viscosity value at very low shear stress. In the case of total hip replacement, the average shear rate ranges from 10^6^–10^7^ 1/s. As a result, synovial fluid has a different viscosity than water. The Cross technique may be used to determine the viscosity in an elastohydrodynamic lubrication study^48^.

7 $$\eta = \eta _{\infty } + \frac{{\eta _{0} - \eta _{\infty } }}{{1 + \alpha (\gamma )^{\beta } }}$$

Since this study uses ANSYS Software, by adapting Eq. (1) and entering the power-law index, then the equation can be re-formulated as follows^49^:

8 $$\eta = H(T)\frac{{\eta _{0} }}{{1 + (\lambda \gamma )^{{1 - n}} }}$$

where *η*_*0*_ is the viscosity at zero shear rate, *η*_*∞*_ is the viscosity of the infinite shear rate, *γ* ˙ is the shear rate (1/s), *λ* is the natural time (the inverse shear rate when the fluid changes from Newtonian to power-law properties), and *n* is the power-law index. Furthermore, the viscosity values applied are *η*_*0*_ = 40 Pas, *η*_*∞*_ = 0.09 mPas, *n* = 0.27, and *λ* = 9.54. Furthermore, Cross^48^ proposed a value of 2/3 for *β*.

### Geometric model

**Figure 1:**
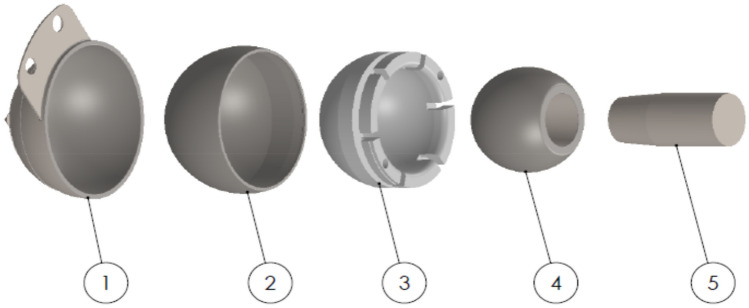
Dual mobility total hip prosthesis components (1. acetabular cup; 2. outer liner; 3. inner liner; 4. femoral head; 5. stem).

**Figure 2:**
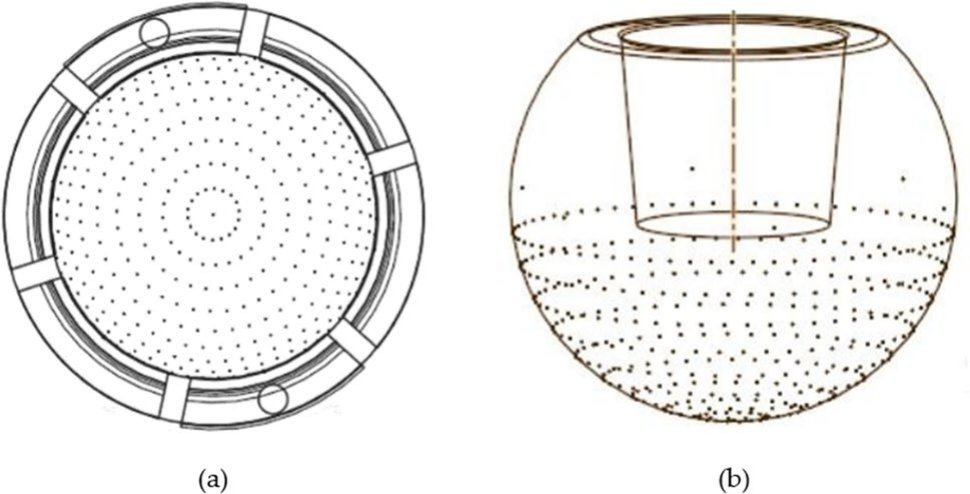
The texture of concave dimple on the artificial hip joint: (**a**) inner liner texture, (**b**) femoral texture.

**Figure 3:**
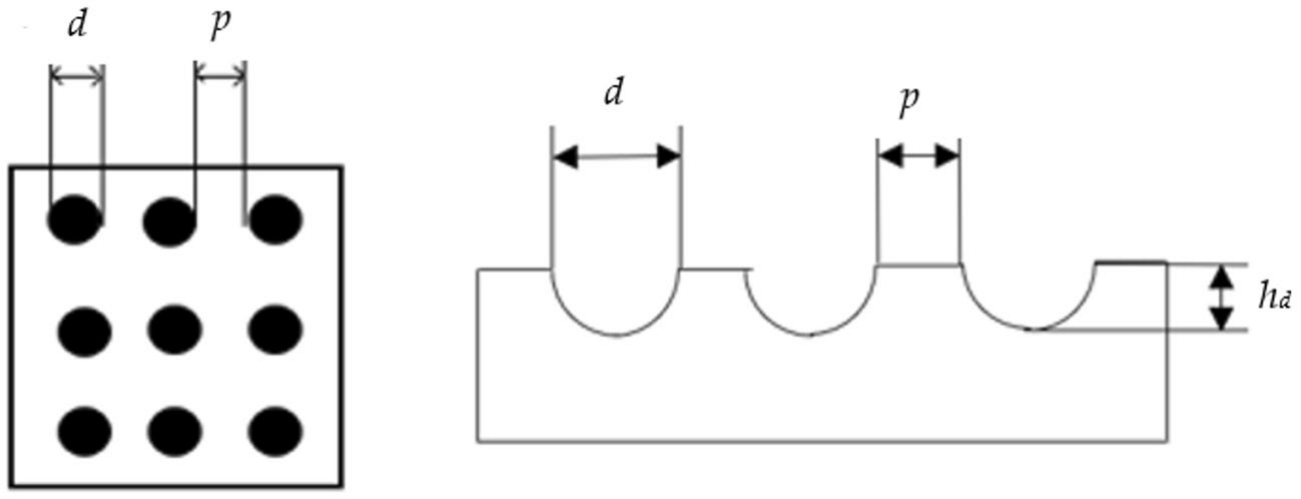
Size of the concave dimple on the femoral head and inner liner. Note: *d* = 0.50 mm, *p* = 0.70 mm,* h*_*d*_ = 0.70 mm.

A dual mobility total hip prosthesis is explored, consisting of ultra-high-molecular-weight polyethylene (UHMWPE) as acetabular cup materials insert to metallic femoral head. In the case of textured bearing, in this work, the concave dimples are applied on femoral head (without texture in liner) and liner (without texture in femoral head) surfaces of dual mobility total hip prosthesis as presented in Fig. 1. In detail, the parameters of dimple geometry are shown in Figs. 2 and 3. Here, *h*_*d*_ denotes the dimple depth (= 0.70 mm), *d* refers to the dimple diameter (= 0.50 mm), and *p* is the distance between the dimples (= 0.70 mm). Due to the apparent identical contact areas on the liner and head, the dimple distribution on the liner surface will be proportional to the synovial fluid produced during Muslim prayer movements. All simulated components in this present study assumed to be homogeneous, isotropic, and linear elastic^50^. Surface roughness in contact interface is not considered into computational modelling^51,52^. To compare the influence of various femoral head materials with dimpled surfaces on either femoral head or liner components, the typically utilized materials for the femoral head, SS 316L and CoCrMo were examined. In detail, Table 1 reflects geometry size, material, and fluid parameters used in the present computation.

**Table 1:** Geometry size, material, and fluid parameters.

Parameter	Size
Geometry^53^	
Femoral head radius	14 mm
Radial clearance (Femoral head to Inner liner)	0.1 mm
Inner liner radius	14.1 mm
Inner liner thickness	6 mm
Outer liner thicness	2.4 mm
Radial clearance (Outer liner to Acetabular cup)	0.1 mm
Acetabular cup thicness	2.4 mm
UHMWPE^38^	
Modulus elasticity	1.1 GPa
Poisson ratio	0.42
SS 316L^38^	
Modulus elasticity	200 GPa
Poisson ratio	0.265
CoCrMo^54^	
Modulus elasticity	230 GPa
Poisson ratio	0.3
Fluid^38^	
Viscosity of synovial fluid at zero shear rate	40 Pa s
Viscosity of synovial fluid at an infinite shear rate	0.9 mPa s
Viscosity power-law index	0.27

**Figure 4:**
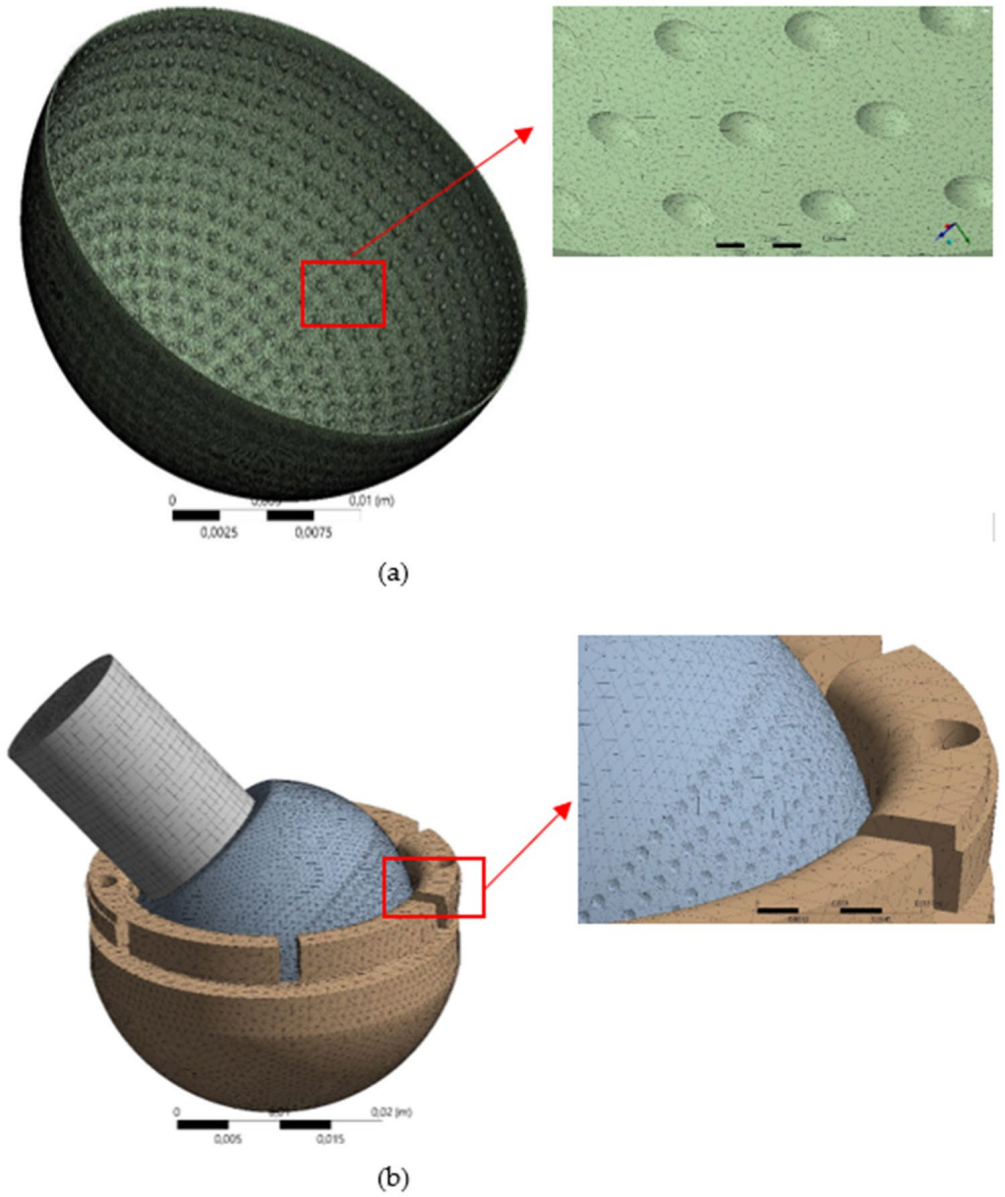
Mesh model on artificial hip joint, (**a**) fluid domain, (**b**) solid domain.

To construct the synovial fluids and components of dual mobility total hip prosthesis, in this work, the 4-node tetrahedral elements are used via ANSYS ICEM-CFD module, which considerably improves computing efficiency and precision. Figure 4 illustrates a three-dimensional meshed model, which is composed of an inner liner, synovial fluid, femoral head, and stem. The sensitivity of the grid density is verified to confirm the accuracy of the results both for the fluid and solid computational domains. To achieve adequate convergence, linear proportional refinement criteria around the dimple area are created for each mesh model for a specific loading situation. Additionally, in terms of computational cost, due to the geometry's complexity, which necessitates a denser element discretization, the sensitivity investigation was carried out under a single loading condition, namely bowing during Muslim prayer movements in this case. The grid size is modified between 800 (coarse mesh) and 80,000 (fine mesh) for the fluid computational domain, and between 80,000 and 790,000 for the solid computational region. The maximum hydrodynamic pressure and maximum von Mises stress values obtained for various grid sizes are depicted in Fig. 4. It should be noted that the maximum von Mises stress profiles shown here are for two different femoral head materials SS 316L and CoCrMo. According to Fig. 4, when the grid size is greater than 45,000 (for the fluid domain) or 520,000 (for the solid domain), the simulation results remain consistent (less than 6%) for both the maximum pressure and the maximum von Mises stress parameter, but the computing cost increases. To summarize, this number of grid systems is used for all simulation situations in the fluid and solid computational domains because it provides an appropriate level of mesh independence while maintaining a manageable computing duration.

In this study, all seven Muslim prayer movements were analyzed with four different models. Rotation around *x*,* y*, and* z* represents flexion–extension, abduction–adduction, and internal–external rotation movements, respectively. The position of the applied load during the Muslim prayer movements was established based on the range of movement, as shown in Table 2, where loading (form as ground rection force) and velocity was explained in Tables 3 and 4, respectively.

**Table 2:** Range of movement during Muslim prayer movements^38^.

Position	Range of movement (°)		
	Flexion (X)	Abduction (Y)	Rotation (Z)
Bowing	87	4	0
Prostration	109.4	6	7 (ex)
Sitting between two prostrations			
Right leg	77.6	6	15 (in)
Left leg	80	4	6 (ex)
Transition from standing to prostration	121.5	0	0
Sitting			
Right leg	78.4	15.5	27.8 (in)
Left leg	74.5	13.2	37.7 (ex)

**Table 3:** Ground reaction force in Muslim prayer (salat) positions expressed as %BW^38^.

Position	GRF (%BW)			Result
	Flexion (X)	Abduction (Y)	Rotation (Z)	
Bowing	0.918	4.295	− 0.21	4.397
Prostration	1.033	3.595	0.043	3.815
Sitting between two prostrations				
Right leg	− 3.409	1.327	− 0.031	3.659
Left leg	1.455	3.097	0.193	3.957
Transition from standing to prostration	0.896	4.221	− 0.326	4.33
Sitting				
Right leg	− 3.758	0.645	− 0.07	3.814
Left leg	− 0.384	3.464	0.163	3.489

**Table 4:** Velocity component in Muslim prayer movemens^38^.

Position	Velocity component		
	Flexion (X) (rad/s)	Abduction (Y) (rad/s)	Rotation (Z) (rad/s)
Bowing	0.759	0	0
Prostration	− 0.106	0.052	− 0.061
Sitting between two prostrations			
Right leg	− 0.278	0	0.192
Left leg	− 0.257	− 0.017	0.009
Transition from standing to prostration	1.061	− 0.035	0
Sitting			
Right leg	− 0.271	0.083	0.304
Left leg	− 0.305	− 0.063	0.391

### Solution setup

To simplify the following numerical simulation, the contact surface with the femoral head is considered a moving wall, and the inner liner is defined as a stationary wall on the contact surface. Because of the elastohydrodynamic nature of the situation, the analysis necessitates a simultaneous solution to the lubricated total hip prosthesis problem. The two-way FSI approach is implemented in this manner. All instances in this study are assumed to be isothermal and the energy equation is not taken into consideration. The second-order upwind technique is employed to discretize the momentum equation. The pressure–velocity coupling is accomplished using the SIMPLE approach.

**Figure 5:**
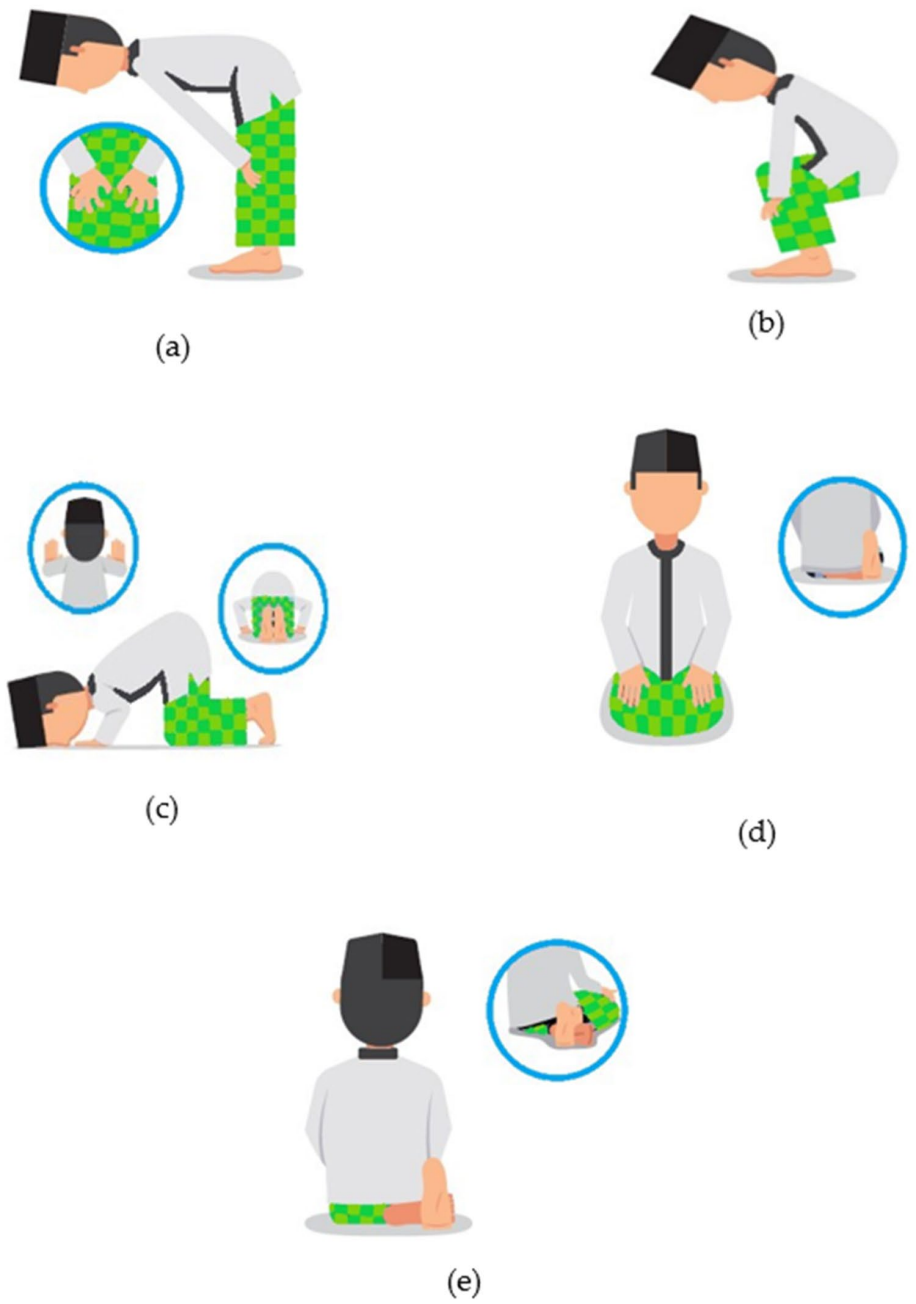
Five intense movements during Muslim prayer (**a**) bowing (*ruku’*), (**b**) transition from bowing to prostration, (**c**) prostration (*sujud*), (**d**) sitting between two prostrations, (**e**) final sitting (*tawarruk*).

In this study, seven hip positions subjected to five intense movements during Muslim prayer namely, (1) bowing (*ruku’*), (2) transition from bowing to prostration, (3) prostration (*sujud*) for the right leg and left leg, (4) sitting between two prostrations, and (5) final sitting (*tawarruk*) for the right leg and left leg, are investigated, as seen in Fig. 5. The applied force is positioned according to range of movement during Muslim prayer movements. Following the work from Wibowo et al.^37^ and Jamari et al.^38^, the velocity load in each position is calculated using the range of motion. The algorithm is built under a variety of loads to establish the ground reaction force (GRF) utilizing the static structure. The GRF is expressed as a % of body weight (percent BW). In this work, the average human body weight was estimated to be 65 kg.

Using ANSYS Workbench, the fluid, and solid equations must be solved simultaneously to investigate elastohydrodynamic lubrication. The system coupling window can be used to achieve the goal. System coupling windows connect the fluid flow (FLUENT) and transient structure windows so that the two-way FSI can be carried out. The establishment of initial conditions for transient structural analysis involves the prior execution of fluid flow simulations using FLUENT software. Subsequently, the obtained fluid flow data is imported into the structural analysis to set the initial conditions. The deformation of the structure leads to alterations in the thickness of the synovial fluid film, consequently impacting the hydrodynamic pressure of the fluid. Solid deformation occurs as a consequence, and this phenomenon persists until the two solutions converge.

### Grid independent test

**Figure 6:**
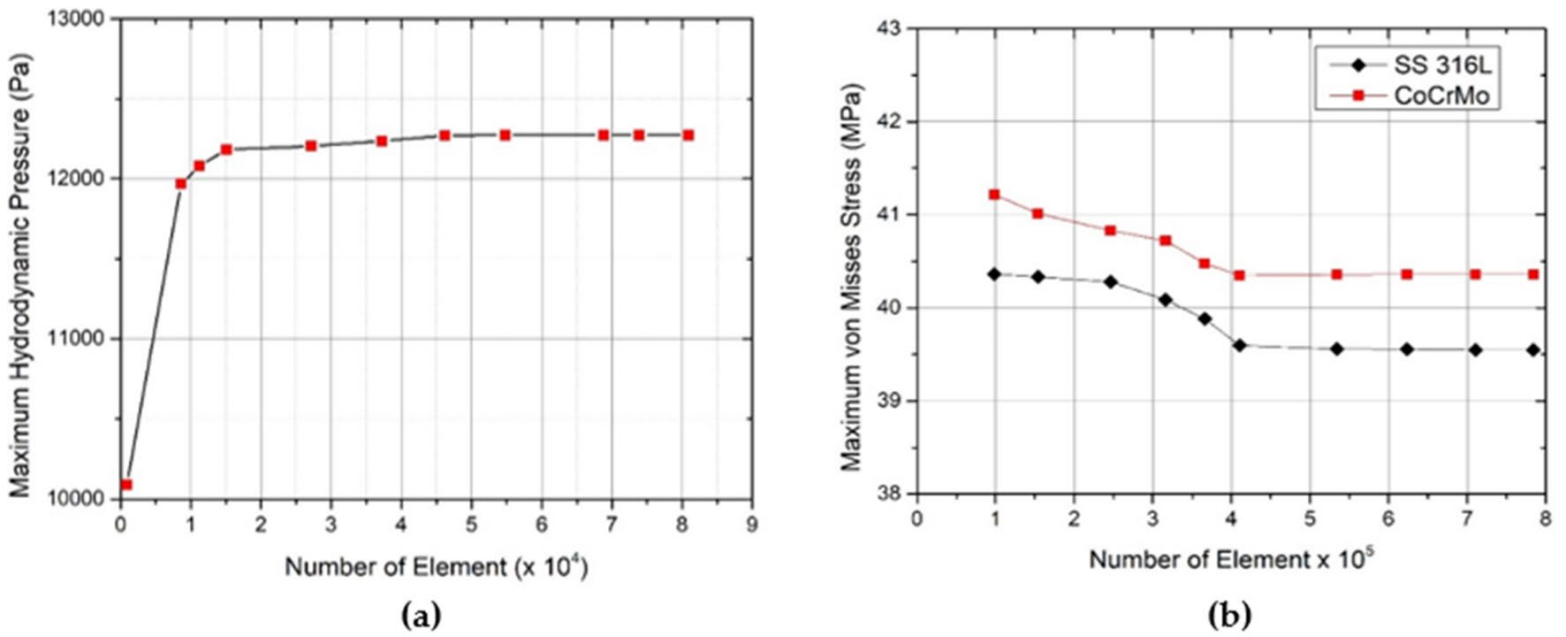
Grid-independent mesh (**a**) hydrodynamic pressure in the fluid domain, (**b**) von Mises stress in the solid domain.

Determination of mesh type and generation density is very important in the simulation to minimize computational time and costs^55^. Generally, mesh generation requires more than half the time required before pre-processing and setting up the discretization of CFD and FEA analysts. The greater the number of mesh elements, the smoother and more accurate the results. However, the larger the mesh element, the longer the simulation process. Therefore, a grid-independent study is conducted to determine the number of appropriate elements and a short time with accurate results. The type of mesh element greatly affects the numerical diffusion, convergence quality, and time. A 4-node tetrahedral element was used to assess the accuracy of the modeling to ensure improved results with a smaller grid size. In addition, a grid-independent study was conducted on the fluid and solid domains as shown in Fig. 6. The calculation predicted hydrodynamic pressure and von Mises stress in the fluid and solid domains, respectively. Furthermore, the computation was conducted using different grid sizes in the two domains with the same initial conditions, namely the transition movement. The grid size varies from 800 (coarse mesh) to 80,000 (fine mesh) and 80,000 to 790,000 in the computation of fluid and solid domains, respectively. Meanwhile, the maximum hydrodynamic pressure and von Mises stress are calculated with the difference in grid size as shown in Fig. 6. The figure shows that there is no difference in the value of hydrodynamic pressure and von Mises stress (below 5%) on grid sizes above 45,000 (in the fluid domain) and 520,000 (in the solid domain). Therefore, it can be concluded that such grids are used in all simulation cases with small sizes, relatively fast computation times, and accurate simulation results.

### Validation

**Figure 7:**
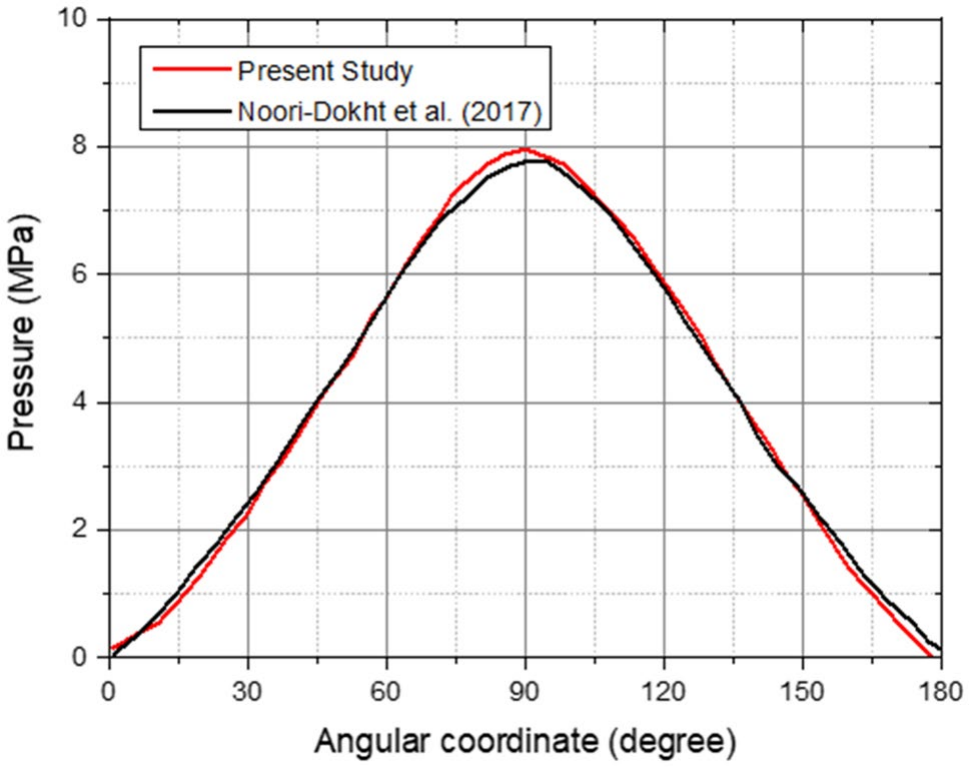
Comparison of hydrodynamic pressure predicted by the present study and Noori-Dokht et al.^57^.

Validation is a term that refers to the process of determining the accuracy of the present simulation findings in comparison to previous studies conducted under the same conditions^56^. Numerical validation is carried out in this study using similar boundary condition values. Additionally, when compared to earlier investigations, the FSI approach accurately represented elastohydrodynamic lubrication with fluid pressure in the transient stage. Figure 7 compares the hydrodynamic pressure values from this investigation to those from Noori-Dokht et al.^57^. According to Fig. 7, the simulation results demonstrate a high degree of resemblance and agreement with the references. However, there is a variance of approximately 5%, which is within the permitted tolerance limits, and these results also validate the simulation method used. In terms of von Mises stress and deformation, results for untextured surface model in the present study have been agreed with previouse study by Wibowo et al.^37^ and Jamari et al. ^38^.

## Results and discussion

**Figure 8:**
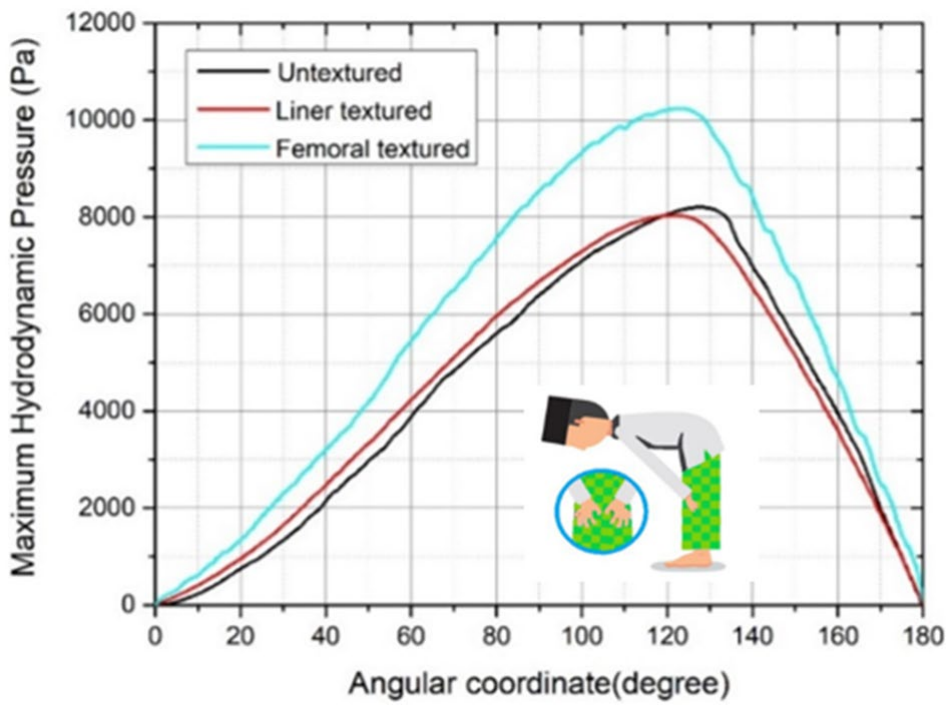
Hydrodynamic pressure distribution for various patterns for the case of bowing during Muslim prayer movements.

**Figure 9:**
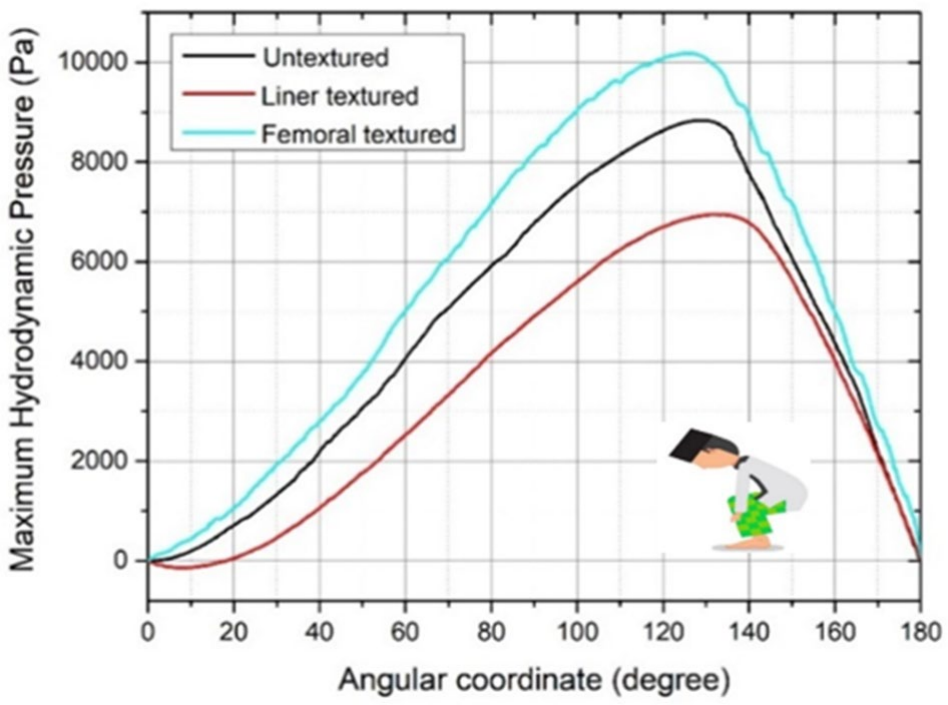
Hydrodynamic pressure distribution for various patterns for the case of transient during Muslim prayer movements.

**Figure 10:**
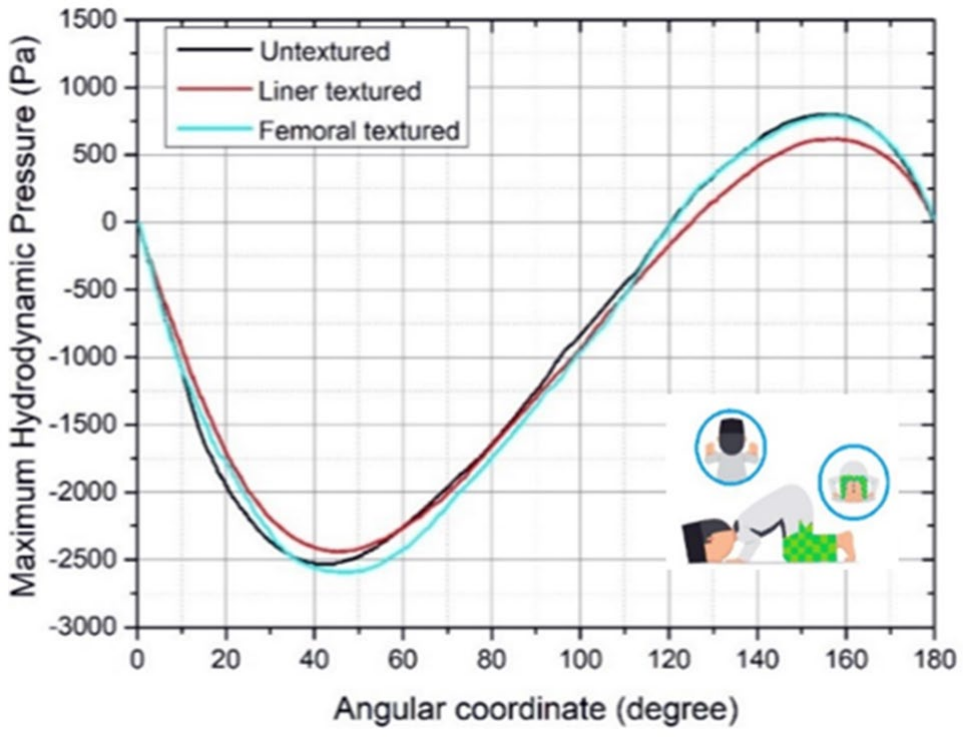
Hydrodynamic pressure distribution for various patterns for the case of prostration during Muslim prayer movements.

**Figure 11:**
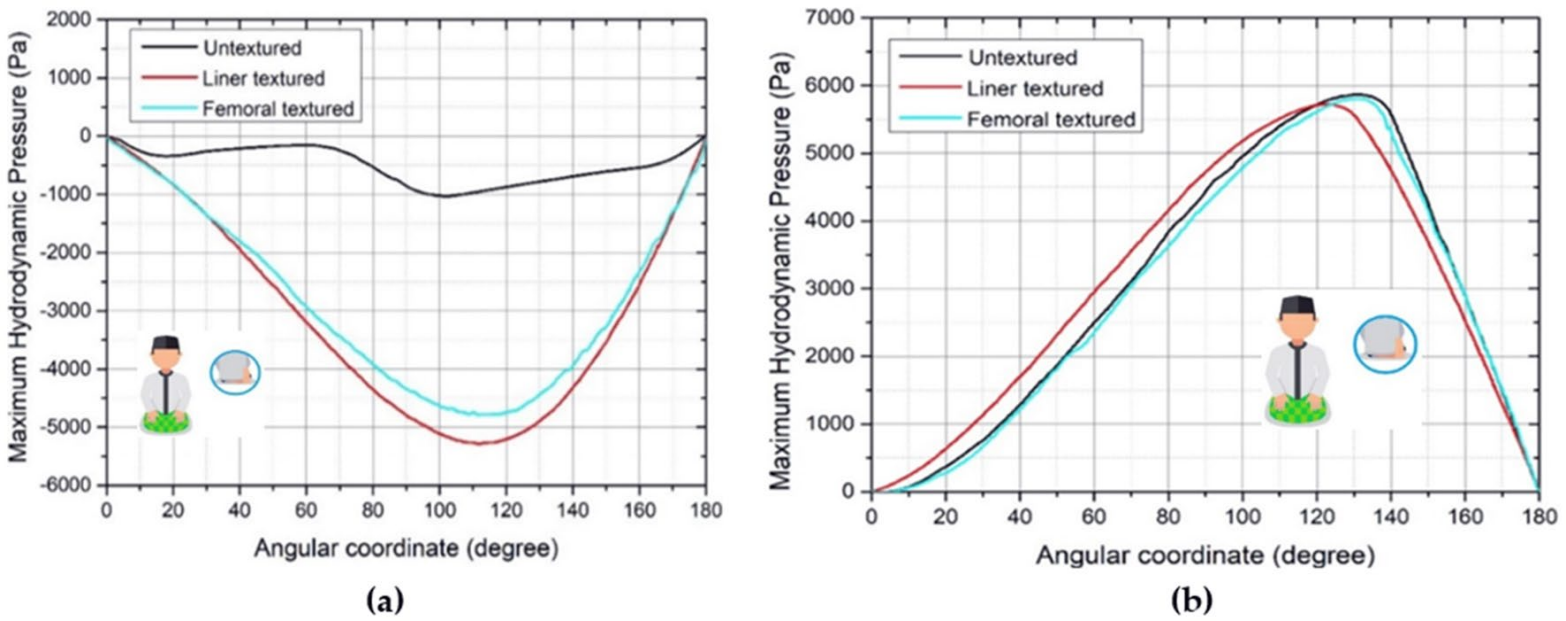
Hydrodynamic pressure distribution for various patterns for the case of sitting during Muslim prayer movements, (**a**) right leg, (**b**) left leg.

**Figure 12:**
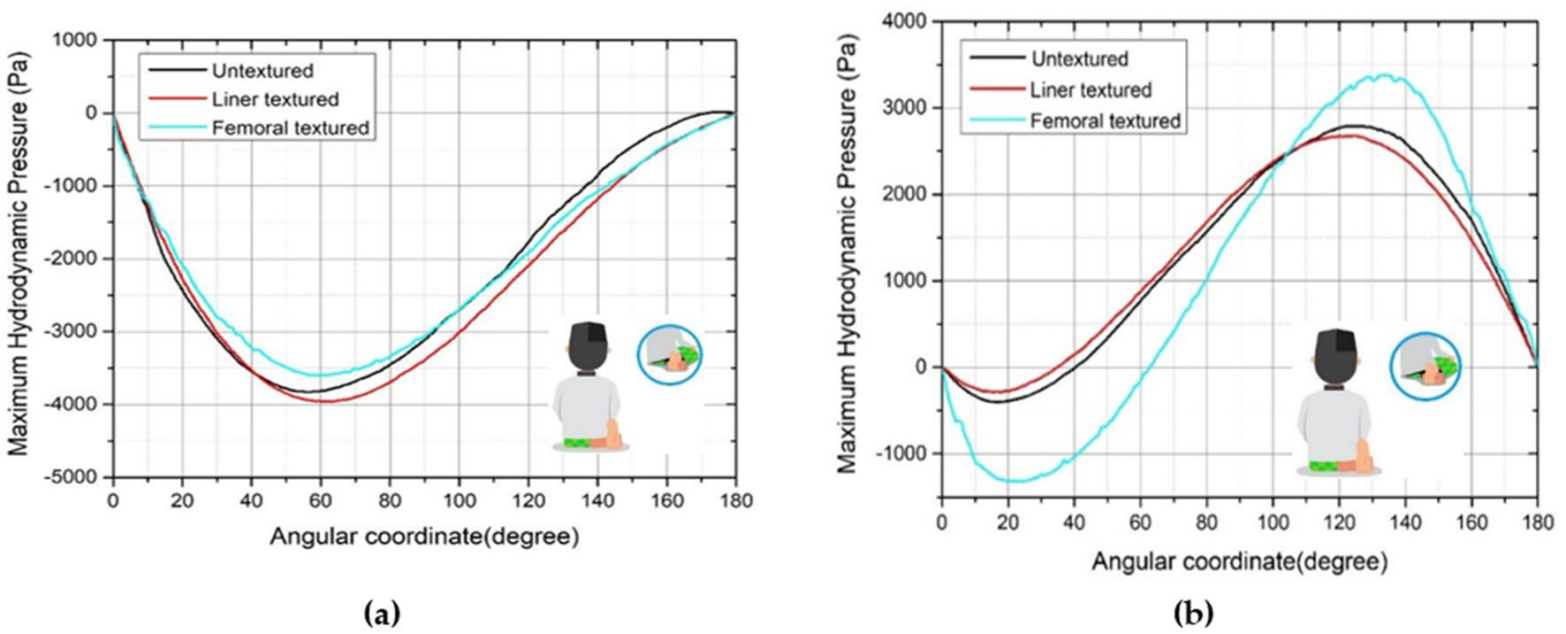
Hydrodynamic pressure distribution for various patterns for the case of final sitting (*tawarruk*) during Muslim prayer movements, (**a**) right leg, (**b**) left leg.

**Figure 13:**
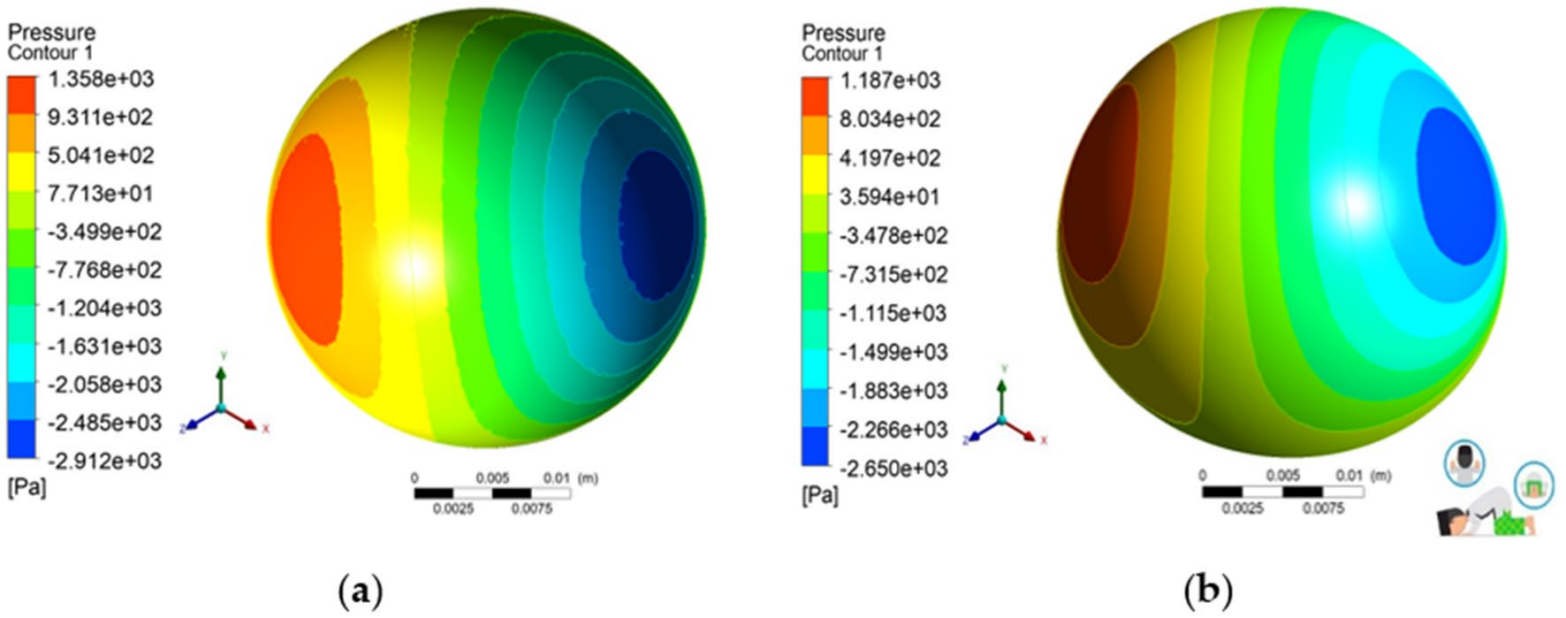
Contour of hydrodynamic pressure distribution during prostration during Muslim prayer movements in the case of (**a**) textured femoral head, (**b**) textured inner liner.

Figures 8, 9, 10, 11 and 12 depict the simulation results in terms of the hydrodynamic pressure in the synovial layer for each movement along an imaginary line ranging from 0° to 180° under different textured patterns. In this study, two texturing cases are of particular interest. The first case involves a textured inner liner, and the second simulates a textured femoral head. For all following computations, all textured results are compared to an total hip prosthesis model with untextured surface model to show the benefits and drawbacks of texturing. Based on Fig. 8, 9, 10, 11 and 12, it can be observed that for each intense movement, different characteristics of pressure are observed. For example, in the case of bowing during Muslim prayer movements, as reflected in Fig. 8, in comparison to other cases, the total hip prosthesis with a textured femoral head provides a superior pressure distribution, as demonstrated by a higher peak pressure. This advantage is also apparent in the case of transition from bowing to prostration during Muslim prayer movements, as illustrated in Fig. 9. It can also be seen based on Fig. 10, that the hydrodynamic pressure changes by 62.77% and 1.002%, respectively when sitting between two prostrations during Muslim prayer movements on the right and left feet at a 120° angle. Meanwhile, when the surface texture model is applied to the prostration during Muslim prayer movements, transition, and sitting (right leg), the hydrodynamic pressure value is reduced in comparison to the untextured surface model. Additionally, Fig. 10a demonstrates that the addition of surface texture to the femoral head and liner results in a decrease of hydrodynamic pressure, which is predominantly negative. Prostration and transition have a maximum difference in hydrodynamic pressure of 1.2% and 16.1%, respectively, when compared to the untextured surface model depicted in Figs. 9 and 10. The contour of hydrodynamic pressure for prostration movement, as illustrated in Fig. 13, reveals that the texture liner results in a higher maximum pressure in comparison to the textured femoral head case. Nevertheless, the magnitude of the deviation is relatively modest, with a discrepancy of only 10%. The existence of a dimple texture on the hydrodynamic pressure contour has a similar pressure distribution value to the surrounding synovial layer's surface area, as indicated by the visible color contour.

**Figure 14:**
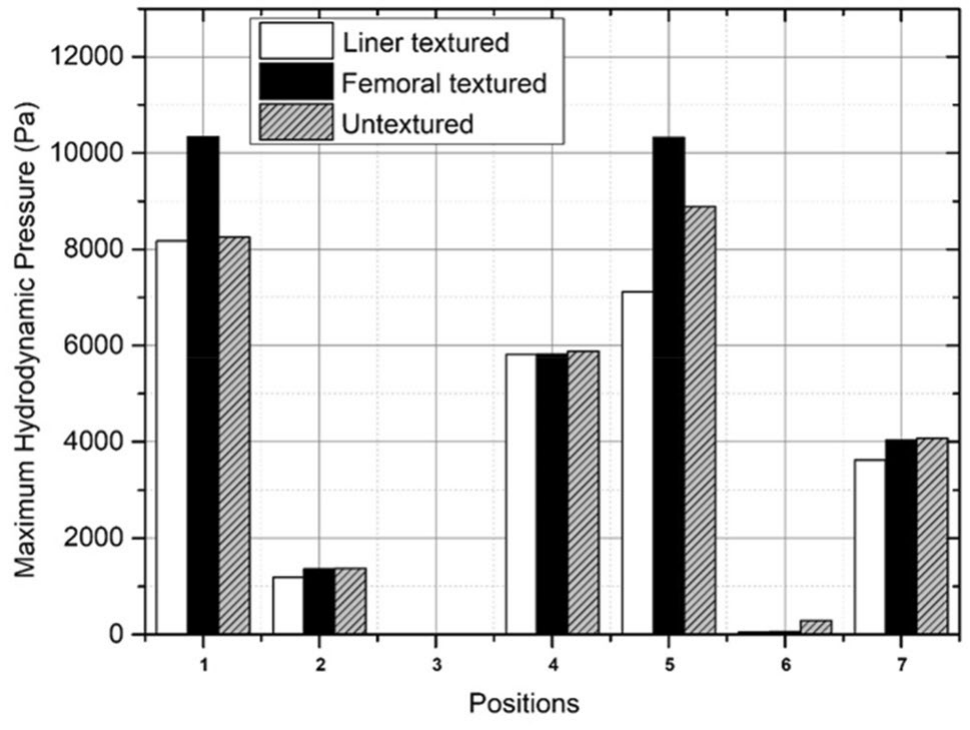
Comparison of maximum hydrodynamic pressure with surface texture variations during Muslim prayer movements. Note: 1-bowing, 2-prostration, 3-sitting between two prostrations (right leg), 4-sitting between two prostrations (left leg), 5-transition, 6-final sitting (right leg), 7-final sitting (left leg).

Based on Tables 5, 6, and Fig. 14, the highest hydrodynamic pressures are sequentially found in bowing, transition, sitting between two prostrations (left leg), and final sitting (left leg) during Muslim prayer movements with textured surface variations. Meanwhile, sitting between two prostrations (right leg), final sitting (right leg), and prostration during Muslim prayer movements have a relatively low maximum hydrodynamic pressure. Regarding the five prayer movements as indicated in Fig. 5, the effect of textured surface can increase the maximum hydrodynamic pressure that occurs in synovial fluid where the variation on femoral head has greatest value as shown in Fig. 14. The effect of textured surface on femoral head and inner liner increases the maximum hydrodynamic pressure up to 25.77% which can be seen in Table 6. This is due to the dimple texture arrangement which acts as a lubrication reservoir when they are filled. Furthermore, each dimple can withstand the perspective of the total load received by the synovial layer^58^. The hydrodynamic pressure value on textured femoral head during Muslim prayer movements is greater compared to textured inner liner. This is because the textured femoral head to be less prone to deformation^59^, therefore making the surface to be maximally filled with synovial fluid^60^.

**Table 5:** Maximum hydrodynamic pressure with surface texture during Muslim prayer movements.

Position	Maximum hydrodynamic pressure (Pa)	
	Textured femoral head	Textured inner liner
Bowing	10,380.40	8170.51
Transition from standing to prostration	10,323.40	7109.60
Prostration	1358.09	1187.52
Sitting between two prostrations		
Right leg	9.90	10.32
Left leg	5819.63	5811.99
Final sitting (*tawarruk*)		
Right leg	59.11	46.08
Left leg	4038.22	3627.19

**Table 6:** Maximum hydrodynamic pressure with and without surface texture during Muslim prayer movements.

Position	Maximum hydrodynamic pressure (Pa)		Percentage (%)
	Untextured surface	Textured surface	
Bowing	8253	10,380.40	25.77
Transition from standing to prostration	8893	10,323.40	16.08
Prostration	1374	1358.09	1.17
Sitting between two prostrations			
Right leg	16.11	9.90	62.77
Left leg	5878	5819.63	1.00
Final sitting (*tawarruk*)			
Right leg	297.7	59.11	403.63
Left leg	4071	4038.22	0.81

**Figure 15:**
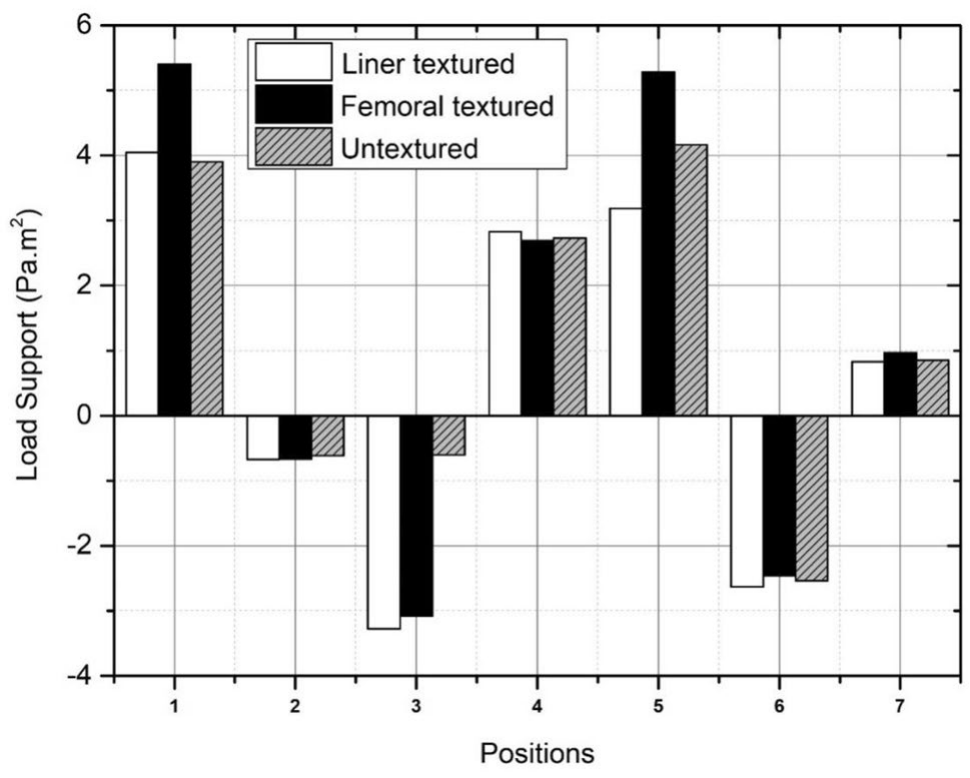
Comparison of load support values with surface texture variations during Muslim prayer movements. note: 1-bowing, 2-prostration, 3-sitting between two prostrations (right leg), 4-sitting between two prostrations (left leg), 5-transition, 6-final sitting (right leg), 7-final sitting (left leg).

The load support calculation is derived by integrating the surface pressure graph curve using ANSYS software. In addition, the computational method employed in the synovial flow simulation incorporates boundary conditions while disregarding the occurrence of cavitation. Consequently, the model accounts for the existence of negative hydrodynamic pressure. Muslim The prayer movements exhibit the highest load support values as a result of textured femoral head variation, as indicated by Fig. 15 and Table 7. The inclusion of dimple on femoral head surface in bowing during Muslim prayer movements giving the most significant outcome compared to other modifications. The results of this study indicate that the increase on textured femoral head side is comparatively more pronounced than on the textured inner liner. The disparity is relatively inconsequential as the texture design currently under investigation has not fully taken into account the design considerations of the synovial layer in terms of its quantity, arrangement, and parameters. In addition, it can be observed from Table 7 that the bowing, sitting (left leg), transition, and final sitting (left leg) during Muslim prayer movements exhibit the highest values among the different texture variations.

**Table 7:** Comparison of load support values with surface texture variations during Muslim prayer movements.

Position	Load support (Pa m^2^)	
	Untextured surface	Textured surface
Bowing	5.40	4.05
Transition from standing to prostration	5.28	3.18
Prostration	− 0.66	− 0.67
Sitting between two prostrations		
Right leg	− 3.08	− 3.27
Left leg	2.69	2.83
Final sitting (*tawarruk*)		
Right leg	− 2.45	− 2.63
Left leg	0.96	0.83

Table 8 shows the comparison of load support values between the textured and untextured surface model in each intense movement during Muslim prayer. The effect of textured surface application on bearing of total hip prosthesis increases the load support compared to untextured surface model. This is indicated by an increase in the value on bowing and sitting between the two prostrations on the left leg during Muslim prayer movements. Meanwhile, in other Muslim prayer movements, textured surface reduces the resulting load support value. Tables 7 and 8 show negative values for the prostration, sitting between two prostrations (right leg), and final sitting (right leg) during Muslim prayer movements. These values indicate that the model cannot provide sufficient load support because the hydrodynamic pressure surface plot is in the negative region. Therefore, the load support for the three Muslim prayer movements is insufficient due to the design of dual mobility total hip prosthesis.

**Table 8:** Comparison of load support with variations in textured and untextured surface during Muslim prayer movements.

Position	Load support (Pa m^2^)		Percentage (%)
	Untextured surface	Textured surface	
Bowing	3.90	5.40	38.44
Transition from standing to prostration	4.16	5.28	26.93
Prostration	− 0.62	− 0.66	7.06
Sitting between two prostrations			
Right leg	− 0.60	− 3.08	410.03
Left leg	2.73	2.69	1.55
Final sitting (*tawarruk*)			
Right leg	− 2.53	− 2.45	3.29
Left leg	0.85	0.97	13.48

**Figure 16:**
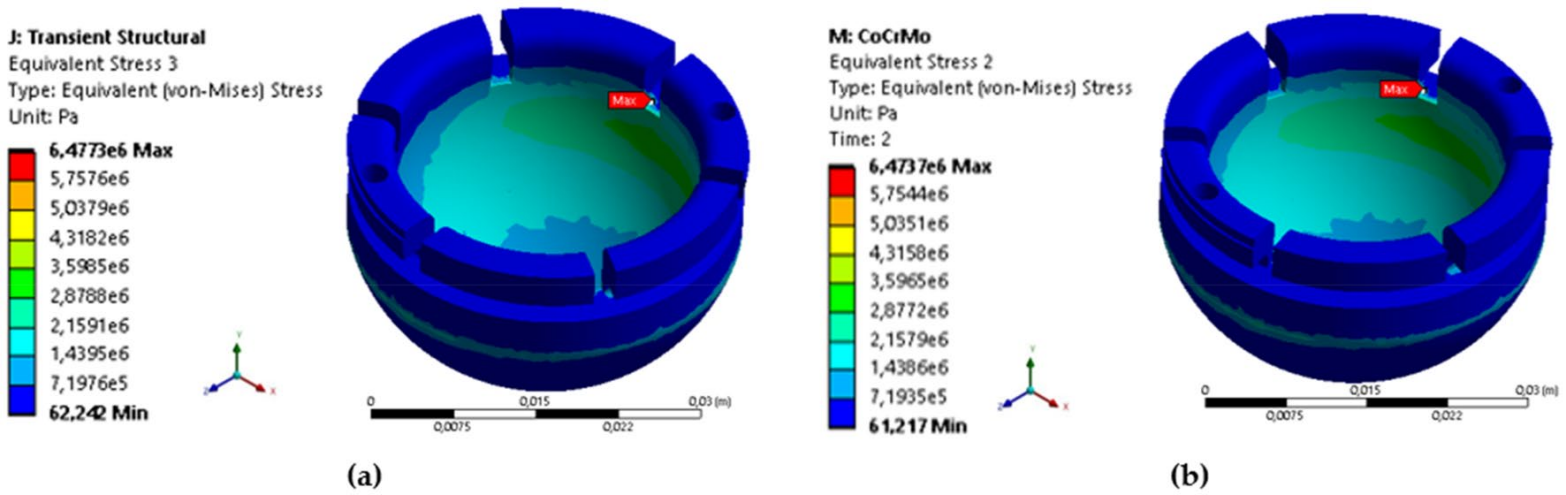
The contour of von Mises stress in sitting between two prostrations (right leg) during Muslim prayer movements: (**a**) SS 316L, (**b**) CoCrMo.

**Figure 17:**
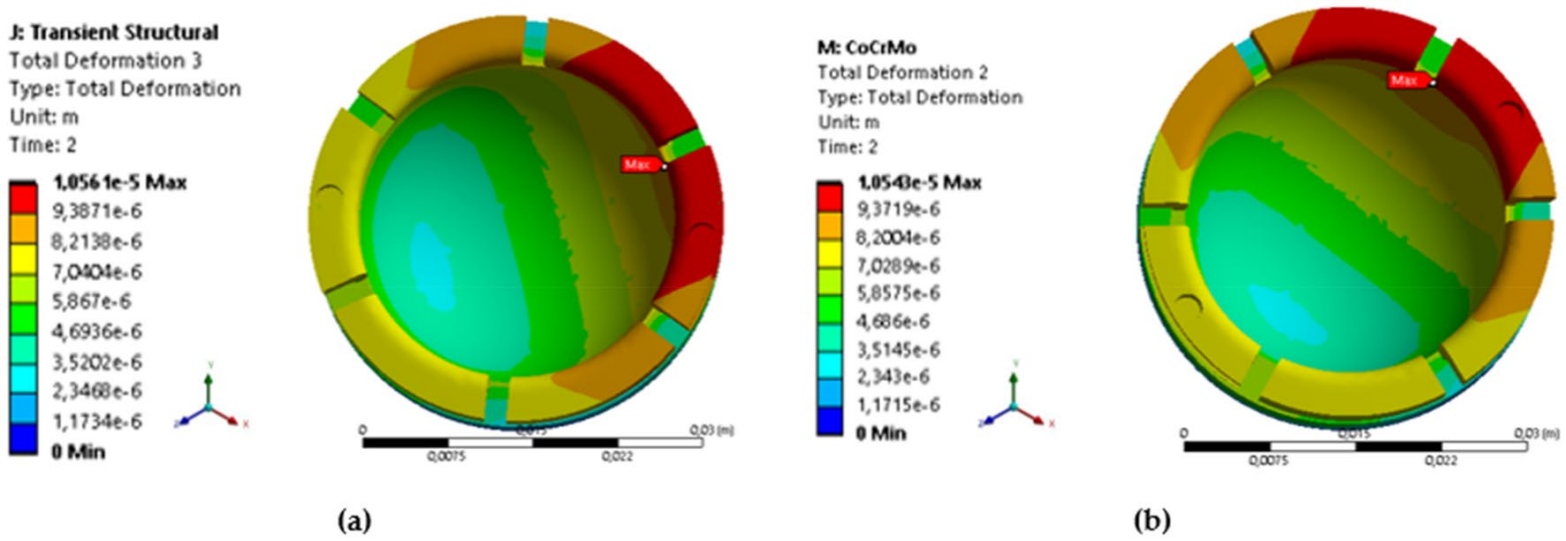
The contour of deformation in sitting between two prostrations (right leg) during Muslim prayer movements: (**a**) SS 316L, (**b**) CoCrMo.

**Figure 18:**
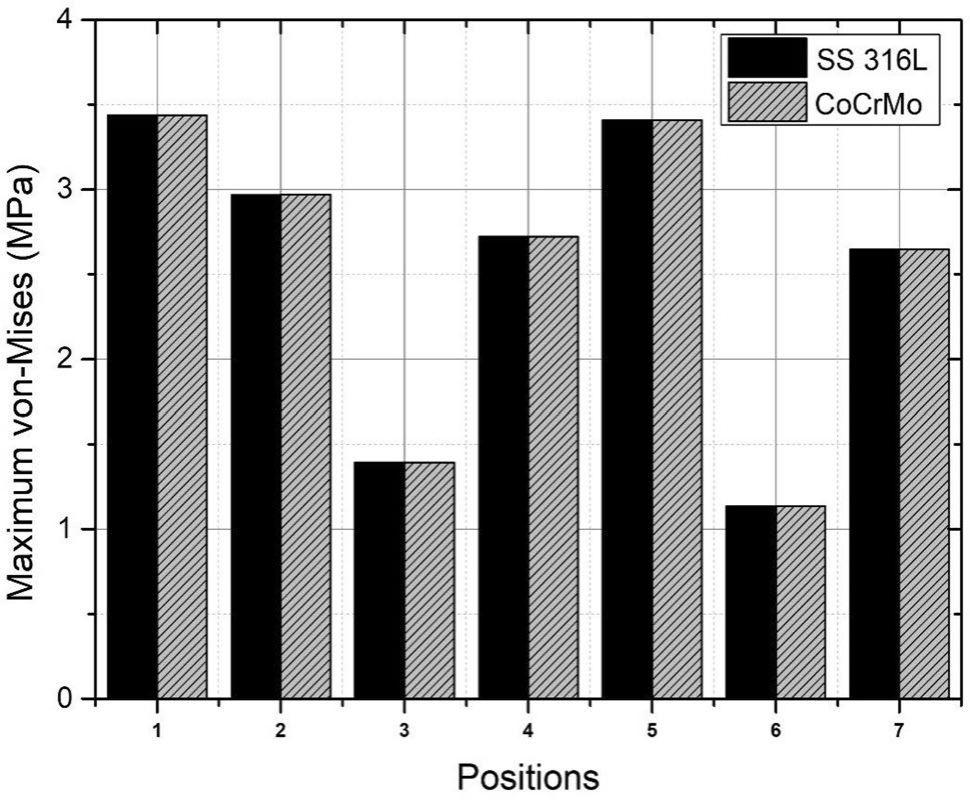
Comparison of von Mises stress on the inner liner with material variations during Muslim prayer movements. Note: 1-bowing, 2-prostration, 3-sitting between two prostrations (right leg), 4-sitting between two prostrations (left leg), 5-transition, 6-final sitting (right leg), 7-final sitting (left leg).

The finite element analysis of the inner liner component made from UHMWPE and femoral head component made from SS 316L and CoCrMo alloy in Fig. 16 (for both materials) indicates that the maximum stress is less than the yield strength of UHMWPE (21 MPa^61^), with a range of 1.1347–3.4377 MPa as shown in Table 9. Additionally, the maximum deformation findings of varying materials indicate that the CoCrMo alloy femoral head is marginally superior to those manufactured of SS 316L as indicated in Fig. 17. This is because the elastic deformation of the components is quite tiny for each prayer movement, less than 0.01 mm. Meanwhile, a study of the maximum von Mises stress across two different materials reveals nearly identical findings for each prayer movement, with a variation of only around 0.001–0.002 MPa, as seen in Fig. 18.

**Table 9:** Comparison of maximum von Mises stress and deformation in the inner liner with material variations during Muslim prayer movements.

Position	Max. von Mises (MPa)		Deformation (mm)	
	SS 316L	CoCrMo	SS 316L	CoCrMo
Bowing	3.4375	3.4377	0.0088	0.0087
Prostration	2.9678	2.9680	0.0117	0.0118
Sitting between two prostrations				
Right leg	1.3938	1.3938	0.0099	0.0098
Left leg	2.7225	2.7226	0.0071	0.0071
Transition from standing to prostration	3.4075	3.4076	0.0096	0.0096
Final sitting (*tawarruk)*				
Right leg	1.1347	1.1348	0.0130	0.0130
Left leg	2.6457	2.6457	0.0113	0.0113

**Figure 19:**
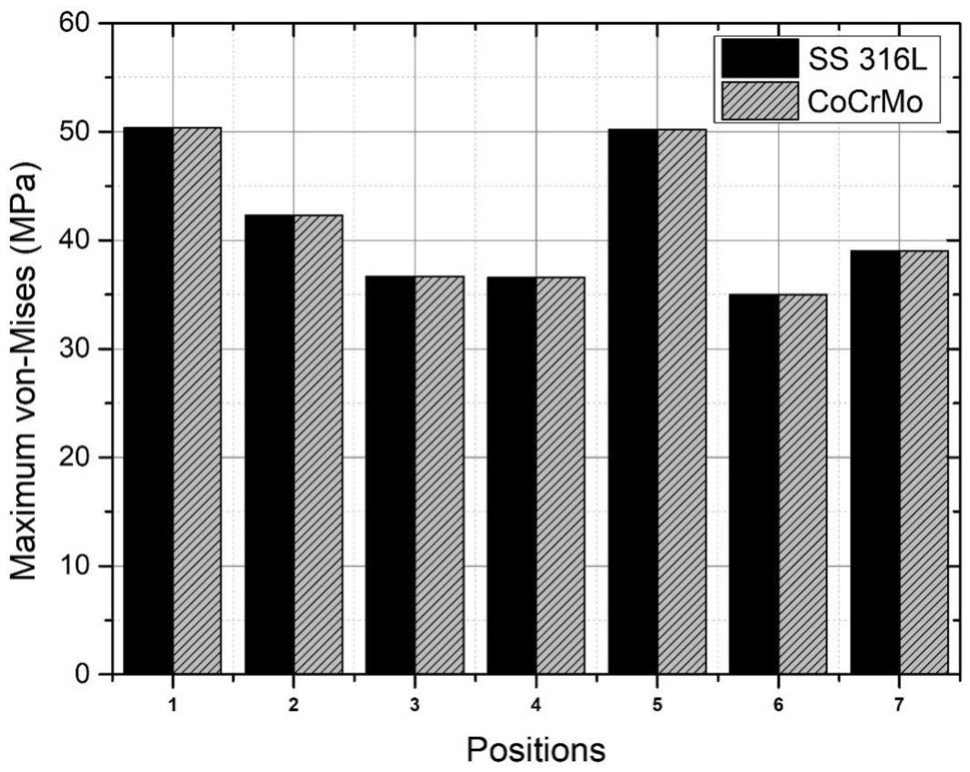
Comparison of von Mises stress on the femoral head with material variations during Muslim prayer movements. Note: 1-bowing, 2-prostration, 3-sitting between two prostrations (right leg), 4-sitting between two prostrations (left leg), 5-transition, 6-final sitting (right leg), 7-final sitting (left leg).

Based on the findings presented in Fig. 19 and Table 10, an examination was conducted to assess the von Mises stress on the femoral head utilizing two different materials. The results indicate that there is no statistically significant disparity in the maximum stress value observed between SS 316L and CoCrMo. The bowing movement exhibits the highest recorded value at 50.35 MPa, yet it remains below materials yield strength of SS 316L (375 MPa^62^) and CoCrMo (612 MPa^63^). In the present study, it has been observed that the magnitude of deformation in SS 316L and CoCrMo alloys is similar, falling within the range of 0.003–0.005 mm. However, it is noteworthy that the CoCrMo alloy demonstrates a relatively lower level of distortion.

**Table 10:** Comparison of maximum von Mises stress and deformation on the femoral head with material variations during Muslim prayer movements.

Position	Max. von Mises stress (MPa)		Deformation (mm)	
	SS 316L	CoCrMo	SS 316L	CoCrMo
Bowing	50.3548	50.3542	0.0089	0.0035
Prostration	42.3143	42.3143	0.0117	0.0117
Sitting between two prostrations				
Right leg	36.6690	36.674	0.0099	0.0099
Left leg	36.5730	36.5735	0.0071	0.0071
Transition from standing to prostration	50.1852	50.1840	0.0095	0.0096
Final sitting (*tawarruk*)				
Right leg	35.0133	35.0153	0.0131	0.0131
Left leg	39.0356	39.0354	0.0113	0.0118

## Conclusions

Two-way FSI modeling is used to simulate elastohydrodynamic lubrication on the performance of dual mobility total hip prosthesis with variation of surface texturing area and femoral head materials. The simulation results show that surface texturing application affects the lubrication performance during Muslim prayer movements. In addition, the textured femoral head model revealed an increase in hydrodynamic pressure compared to untextured femoral head and textured inner liner model. In bowing during Muslim prayer movemens, textured femoral head model increases fluid pressure up to 25.77% compared to untextured femoral head and inner liner model. However, in some Muslim prayer movements such as prostration, sitting between two prostation (right leg), and final sitting (right leg), the pressure decreases compared to that obtained using a untextured surface. This happens because the number, arrangement, and parameters of texture design currently studied have not fully accommodated the design considerations of the synovial layer. The effect of textured surface is very dependent on the simulated loading. Therefore, the effect of surface texturing variations should be studied with the load support results in each of Muslim prayer movements. Moreover, prostration, sitting between two prostrations (right leg), and final sitting (right leg) during Muslim prayer movements has insufficient load support values, which triggers direct contact between femoral head and inner liner. The material variation on femoral head in alaysis of von Mises stress and deformation shows that CoCrMo has a lower deformation than SS 316L. Therefore, CoCrMo femoral head produces better performance in the loading during Muslim prayer movements.

## Data Availability

The data presented in this study are available on request from the corresponding author.

## List of symbols

*c*: Clearance between the femoral head and the acetabular liner
${\mathbf{d}}$: Displacement of the structure domain
${\mathbf{\ddot{d}}}$: Local acceleration of the structure domain
${\mathbf{F}}_{{\mathbf{s}}}$: Body force vector
*h*: Relative rigid displacement
*n*: Power-law index
*p*: Hydrodynamic pressure
*V*: Velocity
*γ̇*: Shear rate
*δ*: Total elastic deformation
*η*_*0*_: Viscosity at zero shear rate
*η*_*∞*_: Viscosity of the infinite shear rate
*λ*: Natural time
*µ*: Dynamic viscosity of lubricant
*ρ*: Density of lubricant
*ρ*_*s*_: Solid density
${{\varvec{\upsigma}}}_{{\mathbf{s}}}$: Solid stress tensor
$${{\varvec{\uptau}}}$$: Stress
